# Development and validation of a scale to evaluate students’ future impact perception related to the coronavirus pandemic (C-19FIPS)

**DOI:** 10.1371/journal.pone.0260248

**Published:** 2021-11-19

**Authors:** Giuseppina Maria Cardella, Brizeida Raquel Hernández-Sánchez, José Carlos Sánchez-García

**Affiliations:** Department of Social Psychology and Anthropology, University of Salamanca, Salamanca, Spain; Aalborg University, DENMARK

## Abstract

During the outbreak of the novel COVID-19 pandemic, economies around the world underwent unprecedented changes, which negatively limited young people’s perceptions of their future. The study aims to describe the development and validation of the 10-item COVID-19 Future Impact Perception Scale (C-19FIPS), a measurement tool to assess future impact perception related to COVID-19, among university students. Inductive and deductive approaches were used at the phase of the scale development process. Exploratory Factor Analysis (EFA), Confirmatory Factor Analysis (CFA) applying two different SEM based analytical methods, covariance-based SEM (CB-SEM) and variance based SEM (PLS-SEM) were used to explore and predict the data. The EFA output generated two dimensions with 10 items. The dimensions are Personal Perception (C-19 PF) and Country Perception (C-19 CF) that reflects the notion of Future Impact Perception related to COVID-19. The result of the CFA confirmed the EFA result. Based on the reliability and validity check results, it is apparent that the scale demonstrates good psychometric properties. Evidence was also provided for convergent and discriminant validity. The study provided a short, valid and reliable measure to assess the impact of the COVID-19 pandemic on college students’ future perceptions. Knowing how external situations influence the world of young people is useful for the development of targeted interventions that favor their well-being and that can support them in situations perceived as uncertain and risky. Limitations and future lines are discussed.

## Introduction

The changes that occurred following the coronavirus pandemic, due to severe acute respiratory syndrome (SARS-CoV-2) in December 2019 in Wuhan, China, deeply affected the social, political and economic structure of the world [1], with negative repercussions on the expectations of the future, especially of the younger ones.

Although other negative global events have hit the world over the centuries with serious health and economic consequences, for example, Ebola disease which caused a loss of $53 billion in the United States [2] and over 11,300 deaths worldwide [3] or SARS which depreciated 1% of China’s GDP causing economic damage of $54 billion worldwide [4], according to many authors, the COVID-19 pandemic has a disastrous scope, causing an unprecedented financial collapse [5–7].

Containment measures taken by governments to limit the spread of the COVID-19 pandemic, for example, social distancing, smart-working, lockdowns and travel restrictions [8] have put enormous pressure on much of the world economy, leading to the reduction of the workforce in all sectors, and the loss of many jobs [9] that severely affected the financial markets by reducing the value of the stock index by up to 10% in 1 day [10].

Although these events can be interpreted as "opportunities", in terms of sustainable economy or green economy (see for example, Oncioiu et al., [11]), the blockade resulting from the COVID-19 pandemic marks the current situation as an acute crisis [12].

Furthermore, the financial markets suffered a sharp decline. According to the study by Shehzad, Xiaoxing and Kazouz [13], in March 2020, United States, United Kingdom, Spain and China recorded a decline of 12.1% to 25.1%. This situation appears even more worrying in a future perspective, especially for the most fragile social groups and for those who enter the world of work for the first time, for example young people.

Some studies have shown the impact that social, cultural and economic changes have on the perception of students’ work [14], negatively distorting young people’s expectations about their future.

This line includes, for example, the results of a report conducted in 112 countries in August 2020, by the International Labor Organization (ILO), which showed that over 70% of young people (aged 18–29 years) were negatively affected by the closure of schools and universities.

According to the report, 38% of students are uncertain about their future career prospects, convinced that the crisis will create further obstacles to their work and extend the transition period from school to work. In addition, among students who combined study and work, one in six young people lost their jobs due to the coronavirus pandemic, while 42% suffered a reduction in income [15].

Historically, the perception of the future has been extensively studied in the field of psychology [16–19], in particular with reference to university students [20–23], and has been analyzed separately in the areas of cognition [17, 18], behavior or motivation [24] and emotion [25], emphasizing the multidimensional nature of the construct [26].

In recent decades, the definition of future time perception has been related to the Social Cognitive Theory (SCT), a learning theory that emphasizes the interaction between personal characteristics, behavior and environment [27] while acknowledging the active role of individuals [28]. Specifically, individual behavior in a certain learning situation influences and is influenced by environmental (or situational) and cognitive/personal factors. Social-cognitive theory explains how people acquire and maintain certain behaviors, also providing the basis for intervention strategies. According to Lewin [29], one of the first exponents in the study of the future time perspective (FTP), we define the perception of future as “the totality of the individual’s views of his psychological future and psychological past existing at a given time” (p. 75), and he stated that the change in FTP is one of the most important facts of development, which regulate behavior and shape the identity of the individual. From this perspective, FTP is closely linked to the experiences that the subject lives in the social and cultural context, as well as to the stage of development in which he finds himself [30].

Corroborating this view, Nurmi [31] defined the FTP as a "motivational space" for individuals, consisting of three fundamental processes, namely, motivation, planning and evaluation. Motivation refers to interests in the future, planning refers to how individuals plan the realization of their interests, and valuation refers to the extent to which individuals expect their interests to be fulfilled.

Although in the literature the perspective of the future time has been associated with the notion of motivation, it should be noted that the FTP construct differs from other motivational constructs. For example, Atkinson’s theory of achievement orientation [32] views a high instrumental value as advantageous for achieving goals in the immediate future, but not for the distant future; furthermore, Malka and Covington [20] provided evidence that students’ FTP is conceptually and empirically separable from perceptions of instrumentality: FTP was a better predictor of school investment than perceived instrumentality. Similarly, FTP and delay of gratification, i.e. the ability to resist a smaller and immediate reward in favor of the larger but later reward, are different constructs [22] with FTP characterized by activity rather than passivity [19]. Finally, FTP theory differs from goal setting theory [33] in that the latter theory lacks the time perspective.

Since the second half of the last century, the notion of the future as "the building site of constructive behavior and human progress" [17] (p. 40) has stimulated research on the FTP construct in the domains of education and work [19, 34–36] as an important predictor of behaviors and attitudes in all ages and culture. Researchers in education have explored the relationships between FTP and educational outcomes such as learning attitudes, academic engagement, and outcomes [37]. In the field of work, researchers linked FTP to decision making and career planning, career choice satisfaction and professional maturity [38]. In general, these studies indicate that individuals differ in the extent to which they think and feel the future and in the amount of effort they make to achieve their future goals.

Empirical evidence has underlined the influence of university students’ future perceptions on their university career, e.g. academic motivation [20, 39] and academic achievements [40]. For example, Shell and Husman [41], examined the relationships between students’ beliefs about the future and their academic performance. The results indicate that beliefs about the future play an important role in motivating students’ achievement and study.

In particular, individuals have difficulty forming beliefs about the future following important rare events [42, 43]. Furthermore, when beliefs are formed, individuals attribute disproportionate weight to more recent events [44], especially when these events are particularly salient [45–47].

These studies highlighted the influence of several factors, such as psychological and personality traits, and socio-cultural factors in shaping the future goals of people in situations of uncertainty [48]. For example, Morselli [49] stressed the importance of social conditions, such as economic crises, in configuring the future as a threat. The results of a study conducted by Ritchie, Cervone and Sharpe [50] with 161 participants from 13 different countries, showed the negative impact of the pandemic on the belief that they can achieve their future goals.

According to the approach of social-cognitive theory [27], the surrounding environment influences the behavior of the individual through personal perceptions. Not all people perceive the same situation with the same degree of severity. Taking these differences into account and understanding them is important to analyze behavioral responses, especially in adverse situations.

From a literature analysis, two fundamental gaps emerged: 1- Current treatment of COVID-19 around the world has focused primarily on infection control, effective vaccines, and treatment cure rate [51, 52], while the psychosocial aspect remains in part strongly underdeveloped; 2- most of the initiatives implemented to support the economies during the COVID-19 crisis seem to affect the current consolidated companies and the existing industrial sectors, little has been done to protect employment and the continuation of the necessary economic activity. Currently, the goal is to protect the present while the future remains uncertain.

The main aim of the current study was to develop a measure of future perceptions related to COVID-19 pandemic consistent with social-cognitive theory [26]. Furthermore, our goal was to take a first step in developing an assessment tool that would be useful in both research and psychological settings. A comprehensive measure of the future impact perception would be useful for informing support planning and assessing the psychosocial effects of Covid-19 pandemic over time. As the research showed that exposure to uncertain and perceived adverse situations has a stronger impact especially among young people [14], the present study used samples of university students to develop the measure of future perceptions related to COVID-19 pandemic across the final phases of the study.

Understanding the perception that people have towards their future, in working and economic terms and trying to contain negative perceptions following the current economic crisis, appears essential both from a scientific and practical point of view, in particular, taking into account recent empirical evidence demonstrating that perceptions and expectations on the macroeconomic environment substantially shape the decisions of the individual [53–57], with repercussions also on psychophysical health [58–62].

We believe that knowing and understanding what people think of the current economic crisis can help political institutions around the world to plan economic recovery programs that look to the future, but also educators and figures in the welfare sector to implement programs to support those who have a more negative perception, with repercussions on psycho-physical health.

The profound and disproportionate impacts that the pandemic is generating on young people give rise to an urgent call for public policy makers to take measures to contain them. It is essential to promote successful career paths through actions such as incentives for hiring young people, training of skills in line with the new realities of the labor market. The time to act is now, otherwise an entire generation representing the nation’s future will be severely affected simply by the misfortune of starting their working life in the midst of the COVID-19 pandemic.

## Materials and methods

### Phase 1: Item generation

According to Churchill [63], a mixed method approach (deductive and inductive methods) was used in the scale development process. In the first step, a literature review was carried out to generated the items, through two widely known databases, Web of Science and Scopus. Keywords used to search for the articles included “COVID-19”, “Coronavirus” AND “Economic* Cris*”, “Financial* Cris*” AND “Future Perspective”, “Future Perception” for the first review and the keywords "COVID-19", "Coronavirus" And "Scala", "Questionnaire" for the second review. The search result yielded 546 documents and 118 documents respectively.

After eliminating duplicate articles between the two databases, to contain any attribution errors, the following inclusion criteria were identified: (i) scientific articles published in peer-reviewed journals, including printed articles, as scientifically valid sources of knowledge (ii) where it was possible to demonstrate studies on the impact of COVID-19 in the economic sector and in a future perspective (first review) or the existence of tools to analyze the coronavirus phenomenon (second review) through the inclusion of words in the titles, in the abstract and/or the author’s keywords, (iii) written in English or Spanish. From our analysis we therefore excluded chapters of books, books, conference proceedings, notes, etc., written in a language other than English and Spanish. This step led to the final result of 65 articles deemed most relevant (42 first revision articles and 23 second revision articles).

From a cross-analysis between the two reviews it emerged that research on the effects of the coronavirus from a socio-economic point of view is still strongly underdeveloped. In fact, several studies pay more attention to the physical and/or psychological consequences related to COVID-19. It should also be noted that the analysis of the literature shows that the few articles that analyzed the impact of the pandemic on the economic sphere were mostly of a conceptual type studies. This result is important because it underlines, at present, the lack of an empirical correspondence, probably because a useful tool is missing to measure the impact of the coronavirus in the future perception linked to the economic sphere.

In our review, some scales measure the psychological consequences of Covid-19 in terms of fears (FCV-19S, see for example Ahorsu et al., [64]), anxiety (Cas, for example Lee [65]), stress (COVID Stress Scales [66]), Risk Appraisal (CORAS, see Jaspal et al., [67]), and worry for contagion (Pre-Covid-19 Scale, [68]).

Therefore, the COVID-19 Future Impact Perception Scale (C-19FIPS) differs from previous ones for two main reasons: 1- most of the measures considered the psychological or physical aspect of the consequences of COVID-19; 2- most of the above scales are one-dimensional, while the scale used in this study features two dimensions (personal and country level), showing its bidimensional character.

**Fig 1:**
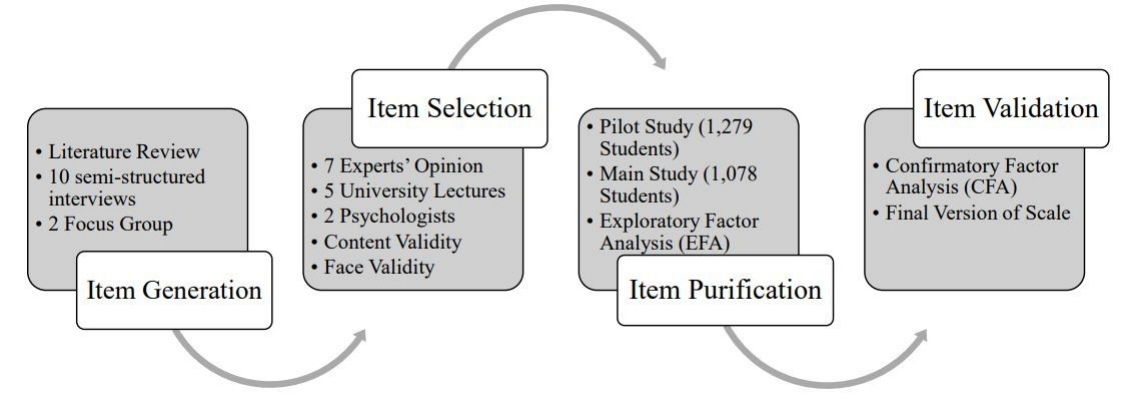
The process followed in this study.

According to Churchill’s [63] suggestion, Fig 1 shows a complete description of the steps carried out for the scale development process.

As a result of a literature review, the C-19 Future Impact Perception was considered as a multidimensional construct consisting of two subsets of elements: the impact on the future perception of work and on the future perception of the country’s economy.

Considering that, as emerged from the literature, there are currently no tools that measure socio-economic perceptions related to COVID-19 in the future, an inductive method has also been used. In this sense, 10 semi-structured interviews were conducted with entrepreneurs and professors of entrepreneurship, in order to obtain feedback on the possible inclusion of some aspects of the construct domain that may not have been included, or suggestions on the exclusion of some other parts of the domain. Furthermore, according to the suggestions of Malhotra et al., [69] two focus groups useful for further brainstorming were carried out. Participants in the first focus group were nine post-graduate students in economics or business science from a public university.

The second focus group was made up of seven researchers from the “Chair of Entrepreneurs” of the University of Salamanca. The choice of homogeneous participants in the focus group is in line with the suggestions of Malhotra et al., [69]. According to the authors, the homogeneity of the interviewees is important because it ensures greater participation and smoother communication, greater self-esteem and less anxiety among the interviewees. Furthermore, homogeneous respondents allow the researcher to make a more accurate theoretical prediction.

With the consent of the respondents, all interviews were recorded and subsequently transcribed. To ascertain the reliability of the information collected, the participants reviewed all transcripts. Before moving on to the next step, a thorough review was carried out to finalize the elements based on the transcripts. The initially created C-19FIPS is a two-dimensional, 24-item, 5-response choice frequency scale.

### Phase 2: Item selection

In the next stage, content validity was performed to understand whether the initial pool of items accurately reflected the C-19 Future Impact Perception construct [70]. In this regard, seven experts were chosen due to their expertise in the subject matter. Five experts were university lecturers specialized in psychometrics and two social psychologists and who were not involved in the study.

In particular, the experts were asked to rate each item on a 3-point Likert scale (e.g.: 1- "essential", 2- "useful but not essential", and 3- "not necessary").

Ten items that did not produce a minimum of a rather representative average value among all the experts were eliminated and 14 items that met the criteria were retained. As suggested by Churchill [63], the expert judges’ recommendations are relevant for the wording reviews, thus helping to establish the validity of the content for the initial C-19FIP Scale.

After the content validity was assured, face validity was assessed by distributing the preliminary questionnaire to 15 post-graduate students. The purpose was to check the questionnaire for timing, clarity of wording, comprehension, layout and style, completeness and flow of control. In addition, respondents were asked to provide their feedback regarding the content, structure and formatting of the questionnaire. Minor suggestions from respondents were considered, which helped to improve the clarity and readability of the questionnaire.

### Participants and procedure

An important issue in the scale development and validation process is the sample size [71, 72]. In the literature, the best and most common rule concerns a ratio of respondents to elements of 10:1, i.e. 10 participants for each element of the scale [73]. However, other authors have suggested sample sizes independent of the number of items. For example, according to Guadagnoli and Velicer [74] the minimum number of participants required for the generalizability of the results is 300–450. Comrey and Lee [75] suggested a graduated sample size scale: 100 = poor, 200 = fair, 300 = good, 500 = very good, ≥1,000 = excellent.

In the present study we used two different sample of undergraduate students (Study 1: n = 1,279; Study 2: n = 1,078)

The demographic characteristics of two sample (Phase 3 and Phase 4) are shown in Table 1.

**Table 1:** Demographics characteristics.

Characteristics	Sample Phase 3 (n = 1,279)	Sample Phase 4 (n = 1,078)
Gender—n (%)		
Male	631 (49.3%)	590 (54.7%)
Female	648 (50.7%)	488 (45.3%)
Age–mean (SD)	22.54 (4.53)	20.54 (2.47)
Academic Course–n (%)		
First-year course	165 (12.9%)	250 (23.2%)
Second-year course	227 (17.7%)	169 (15.7%)
Third-year course	646 (50.5%)	490 (45.5%)
Fourth-year course	209 (16.3%)	138 (12.8%)
Fifth-year course	32 (2.5%)	31 (2.9%)
Type of study–n (%)		
Social Sciences	328 (25.6%)	269 (25.0%)
Health sciences	308 (24.1%)	213 (19.8%)
Humanities	476 (37.2%)	356 (33.0%)
Economics and Business science	167 (13.1%)	240 (22.3%)

Participants come from different fields of knowledge and from different Spanish universities. The selection of different degree courses and universities was made on the basis of completely random samplings and the students participated in the development of the research anonymously.

The online survey data was collected through an internet platform between September/December 2020 with Spanish university students. This period corresponded to the second wave of the COVID-19 pandemic in Spain and students were suffering the psychological consequences of the pandemic, with enormous social restrictions.

Participants were provided with full information on the purposes of the study and the confidentiality of the data assuring them that it would be used for research purposes only. Their participation was on a voluntary basis and any refusal to participate would not affect their current or future course of study. Students completed the questionnaire in approximately 10 min. and received no credit for participating in the research.

The study was performed in accordance with the 1964 Helsinki Declaration and its subsequent amendments, or with comparable ethical standards. Written informed consent was obtained from each student prior to participating in the study. Ethical review and approval was not required for the study on human participants in accordance with the local legislation and institutional requirements, as no intervention was carried out.

Every precaution has been taken to protect the privacy of the research subjects and the confidentiality of their personal information.

### Instruments

The questionnaire included a section for demographic information (i.e., gender, age, degree program, university) and the Future Time Perspective Scale administered to analyze the nomological network and predictive validity of the C-19 Future Impact Perception Scale.

The Future Time Perspective Scale [76] is a 12-item questionnaire assessed on a five-point Likert scale (1 = strongly disagree, 5 = strongly agree). It measures three perspectives of the future: Future as Open (4 items), Future as Limited (4 items) and Future as Ambiguous (4 items). Cronbach’s alpha for the three dimensions in the current study was 0.85, 0.74, and 0.80, respectively.

Examples of Future Time Perspective Scale are: "I look forward to the future with hope and enthusiasm" (Future as Open), " Increasingly I feel like time is against me" (Future as Limited) and “I do not focus on the future because it is so uncertain to me anyway” (Future as Ambiguous).

### Statistical analysis

Descriptive statistics were used to understand participants’ demographic characteristics.

To extract the dimensions of the C-19FIP Scale, Exploratory Factor Analysis (EFA) was used. All the descriptive analysis and EFA were conducted using IBM SPSS 24.0.

In this study the general validity of the model was carried out using the structural equation modeling approach (SEM) and applying the CFA method of comparing two models: a first order model (measurement model) and a second order (structural model).

In the first order model, we applied two different analytical methods based on structural equation models, covariance-based analysis (CB-SEM) and variance-based analysis, know as partial least squares (PLS-SEM), using two different software: Amos 23.0 and SmartPLS 3.0 [77].

Specifically, we used the variance-based method (PLS-SEM) as it focuses on optimizing the prediction of endogenous constructs and the covariance-based (CB-SEM) method for model fitting [78, 79]. For this reason, the choice to use both methods (CB and PLS) must be considered complementary rather than competitive.

Furthermore, the PLS-SEM path modeling was carried out at the structural level to estimate and evaluate the structural properties of the research model, following the procedures suggested by Hair et al. [80].

This choice was made for several reasons. First, in this study, the measurement model that reflects the relationship between the subscales and the respective indicators, and between the second-order construct and the two subscales, was identified as reflective-formative model (Type II).

In particular, at the first order, the direction of causality can be seen from the subscale to the indicators. The indicators are in fact interchangeable and correlated, so the release of an element does not affect the meaning of the subscale. At the second order, the two subscales (C-19 PF and C-19 CF) are distinct constructs because they possess different conceptualizations. Furthermore, these constructs are not highly correlated or interchangeable. Furthermore, a modification of one of these constructs would modify the meaning attributed to the higher-order variable (C-19 Future Impact Perception) [81, 82].

When a structural equation model includes formative variables, the variance-based method (PLS-SEM) provides more accurate estimates than the covariance-based methods (CB-SEM). In agreement with many empirical evidences [83, 84], the failure to use the PLS method for the evaluation of an SEM that includes a formative measurement model can distort the estimates.

Furthermore, PLS-SEM provides a more reliable estimate of a relatively complex model that has reflective (e.g. C-19 PF and C-19 CF) and formative (e.g. C-19 Future perception) measurement models [84, 85].

Finally, PLS-SEM is a non-parametric statistical approach, so it does not require data to be normally distributed [83]. However, it should be noted that although it does not require that the data have a normal distribution, it is necessary to verify that the data is not excessively non-normal, as, in general, this type of data is problematic in evaluating parameters. It is important to specify that the asymmetry and kurtosis values between -2 and +2 are considered acceptable [86].

## Results

### Phase 3: Item purification

Exploratory Factor Analysis (EFA) using principal component analysis with varimax and the Kaiser normalization and rotational method [87] was carried out to investigate the dimensional structure of the questionnaire.

The assessment of factorability showed that the Kaiser–Meyer–Olkin measure was 0.883 and Bartlett’s test of sphericity was significant (x^2^ = 7210,581; df = 45, p< 0.001) indicating that the data were adequate for the factor analysis. The results confirmed the existence of two factors associated with the proposed theoretical arguments of this study. The selection of the extracted factors was decided on the basis of two different criteria: only factors with an eigenvalue greater than 1 and elements that had a factor loading greater than 0.70 [88].

For this reason, items CovidC6 “Covid-19 will negatively affect entrepreneurs in my country” (factor loading = 0.658), CovidC7 “Covid-19 will prevent the creation of new businesses in my country” (factor loading = 0.430), CovidC8 “COVID-19 will diminish labor opportunities in my country” (factor loading = 0.586) and CovidP6 “COVID-19 has discouraged me to become an entrepreneur” (factor loading = 0.557) have been eliminated.

The two-component solution explained a variance of 69.779% from a total of 10 items (Table 2).

**Table 2:** Results of Exploratory Factor Analysis (EFA) (sample n = 1.279).

Items	Factor 1	Factor 2	Communalities
CovidP1	0.857		0.742
CovidP2	0.844		0.734
CovidP3	0.766		0.616
CovidP4	0.865		0.754
CovidP5	0.867		0.759
CovidC1		0.823	0.678
CovidC2		0.861	0.751
CovidC3		0.851	0.737
CovidC4		0.756	0.584
CovidC5		0.738	0.622
Eigenvalue	3.646	3.332	
Variance	36.459%	33.320%	

The first component (5 items, explained variance = 36.459%) was loaded by items referring to the perception of Covid-19 linked to relationships with one’s future work (C-19 PF).

The second component (5 items, explained variance = 33.320%) was loaded by items related to the perception of Covid-19 in the economic sphere of one’s country (C-19 CF).

### Phase 4: Item validation

#### First-order measurement model

To assess the convergent validity of the first-order constructs, a PLS algorithm with factor weighting scheme and 300 iterations were run to generate the values of factor loadings, composite reliability (CR), and average variance extract (AVE).

According to Hair et al. [78], the factor loading of each items on the respective construct should be greater than 0.70. Loadings between 0.40 and 0.70 can be removed if they lead to an improvement in the model.

Furthermore, if the values of CR and AVE of each factor are higher than 0.70 and 0.50, respectively, the questionnaire is considered as having a satisfactory convergent validity [89, 90].

Composite reliability (CR), Cronbach’s alpha (CA) and coefficient Dijkstra-Henseler Rho_A are the measures of internal consistency reliability and refer to the extent to which a construct is measured by its relevant indicators [90]. As shown in Table 3, the factor loadings of all observed variables exceeded the threshold value of 0.70. Moreover, the CR, CA and Dijkstra-Henseler reliability for latent variables surpassed the cut-off value of 0.70. Thus, the reliability of all measures was assured at the item and construct level.

**Table 3:** First-order measurement model.

Dimensions	Items	Factor Loadings	Cronbach’s Alpha >0.70	rho_A >0.70	Composite Reliability >0.70	AVE >0.50
	CovidP1	0.839				
	CovidP2	0.853				
C-19 PF	CovidP3	0.759	0.891	0.893	0.920	0.699
	CovidP4	0.866				
	CovidP5	0.857				
	CovidC1	0.784				
	CovidC2	0.848				
C-19 CF	CovidC3	0.850	0.872	0.876	0.907	0.662
	CovidC4	0.771				
	CovidC5	0.811				

The assessment of construct validity was confirmed based on convergent validity and discriminant validity [91]. Convergent validity refers to the extent to which a set of indicators that measure the same construct are positively correlated. The dominant measure of convergent validity is the AVE [89].

An AVE value greater than 0.50 is considered as acceptable [89]. As demonstrated in Table 3, AVE values for constructs transcended the advised value of 0.50. Thus, convergent validity was assured.

Discriminant validity refers to the extent to which a construct is empirically distinct from other constructs in the model. Fornell and Larcker [89] proposed the traditional metric and suggested that each construct’s AVE should be compared to the squared inter-construct correlation of that same construct and all other reflectively measured constructs in the structural model. The shared variance for all model constructs should not be larger than their AVE values. Recent research indicates, however, that this metric is not suitable for discriminant validity assessment. For example, Henseler et al. [92] show that the Fornell-Larcker criterion does not perform well, particularly when the indicator loadings on a construct differ only slightly. As a replacement, Henseler et al. [92] proposed the Heterotrait-Monotrait ratio of the correlations (HTMT). The HTMT is defined as the mean value of the item correlations across constructs relative to the mean of the average correlations for the items measuring the same construct. An HTMT value above 0.90 would suggest that discriminant validity is not present. In addition, bootstrapping can be applied to test whether the HTMT value is significantly different from 1.00 [92] or a lower threshold value such as 0.85 or 0.90, which should be defined based on the study context [93]. More specifically, the researcher can examine if the upper bound of the 95 per cent confidence interval of HTMT is lower than 0.90 or 0.85.

According to the first criteria, the square root of AVE should be higher than the correlation with all other variables in the model. Table 4 revealed that the square root of AVE values (C-19PF AVE = 0.834; C-19CF AVE = 0.805) are higher than the correlation values (correlation between county and personal dimension: r = 0.256, p< 0.001).

**Table 4:** Fornell–Larcker criterion.

	C-19 PF	C-19 CF
**C-19 PF**	(0.836)	
**C-19 CF**	0.225	(0.814)

**Note:** The elements on the diagonal values in parentheses represent the square root of the variance extracted (AVE) and the values outside the diagonal represent the correlations between the factors.

On the other hand, the second criteria estimate, the Heterotrait-Monotrait ratio of Correlations (HTMT) values should be less 0.90, as recommended by Henseler et al. [92]. In addition, the bootstrapping procedure with 5000 samples is performed to demonstrate additional evidence that HTMT value is significantly different from 0.90. In our study the confidence interval of HTMT does not include 1 (HTMT = 0.247; C.I. [0.176–0.310]). Overall, discriminant validity can be accepted for this measurement model.

To test the model fit, the Standardized Root Mean Square Residual (SRMR) [94] was used: a value of 0 for SRMR would indicate perfect fit and, in general, an SRMR value less than or equal to 0.05 indicates an acceptable fit [95]. A recent simulation study shows that a specified correct model implies SRMR values greater than 0.06 [96]. In this study, the SRMR was 0.07, indicating a good fit of the model.

A replication of the confirmatory analysis was performed based on the covariance-based SEM (CB-SEM) approach and maximum likelihood estimation, using the AMOS 23.0 software.

The most common fit indices were used to fit the model. Goodness of fit indices were used when the ratio between the chi-squared value and the degree of freedom was lower than 3 (X^2^/df), the comparative fit index (CFI) and Tucker–Lewis index (TLI) (acceptable fit: ≥ 0.95), the Root Mean Square Error of Approximation (RMSEA) was also included as a goodness of fit index and it must be lower than 0.05 or assumes a maximum value of 0.08 [97].

The two-factor model demonstrated a good fit: X^2^ = 249,749 (85), p< 0.001, CFI = 0.962, TLI = 0.950 and RMSEA = 0.07. Furthermore, as Fabrigar et al. [98] recommend, the correlation between the two dimensions is less than 0.70 (r = 0.224), supporting the good fit of the measurement model.

However, as Table 4 shows Confirmatory Factor Analysis (CB-SEM) revealed valid scores of the factor loadings, but with lower values than the factor loadings generated by the PLS-SEM analysis. In this sense, according to Afthanorhan [99], we consider the PLS-SEM approach as a more accurate and precise method. As the author explains, in fact, the modeling of the PLS-SEM pathway is more appropriate to perform the most reliable and valid CFA, since it maximizes the explained variance of the endogenous latent constructs (dependent variable).

The non-standardized and standardized parameter estimates are all provided in Table 5 and are statistically significant (p< 0.01). The R^2^ values show the amount of variance of the elements explained by their respective constructs.

**Table 5:** Result of CFA CB-SEM.

Items	Unstandardized Estimate	Standardized Estimate	R^2^	S.E.	C.R.	*p*
CovidP2	1.018	0.807	0.65	0.34	30.257	**
CovidP3	0.951	0.801	0.64	0.32	29.944	**
CovidP4	0.725	0.669	0.45	0.31	23.586	**
CovidP5	0.965	0.836	0.70	0.30	31.725	**
CovidP6	1.000	0.832	0.69			
CovidC1	0.643	0.740	0.55	0.28	23.034	**
CovidC2	0.938	0.826	0.68	0.37	25.542	**
CovidC3	0.975	0.814	0.66	0.39	25.219	**
CovidC4	0.849	0.692	0.48	0.39	25.124	**
CovidC5	1.000	0.731	0.53			

** p< 0.001

Convergent validity is assessed through computing the Average Variance Extracted (AVE) using a factor loading from each construct. For PLS-SEM, the minimum requirement for indicator loading in the model is 0.70 because the square of that value is equivalent to 50% of the variable variance. The minimum factor loading for CB-SEM is 0.50, and the item deletion should not exceed 20% of the total indicators in the model [80]. In our model, the AVE is of 0.626 and 0.581 respectively, indicating that, for both the two dimensions, more than 50% of the variance of the indicator is explained in the construct score.

Discriminant validity is the last step for establishing the validity of measurement model. The constructs in a model must be discriminant to each other in order to avoid the problem of multicollinearity [100]. The approach most used by researchers to establish discriminant validity is to compare the square root value of AVE with correlations between two constructs. As shown in Table 6, the square root of the AVE of each construct is greater than the correlation between the constructs. The discriminant validity requirements are satisfactory.

**Table 6:** Convergent and discriminant validity CB-SEM.

Dimensions	AVE	Cronbach’s Alpha	Composite Reliability	C-19 PF	C-19 CF
C-19 PF	0.626	0.892	0.893	**0.791**	
C-19 CF	0.581	0.865	0.874	0.224	**0.762**

**Note.** The bold number is the square root of AVE. The bold numbers listed diagonally are the square root of the variance shared between the constructs and their measures. The off-diagonal elements are the correlations among the constructs. For discriminate validity, the diagonal elements should be larger than the off-diagonal elements.

#### Second-order measurement model

**Fig 2:**
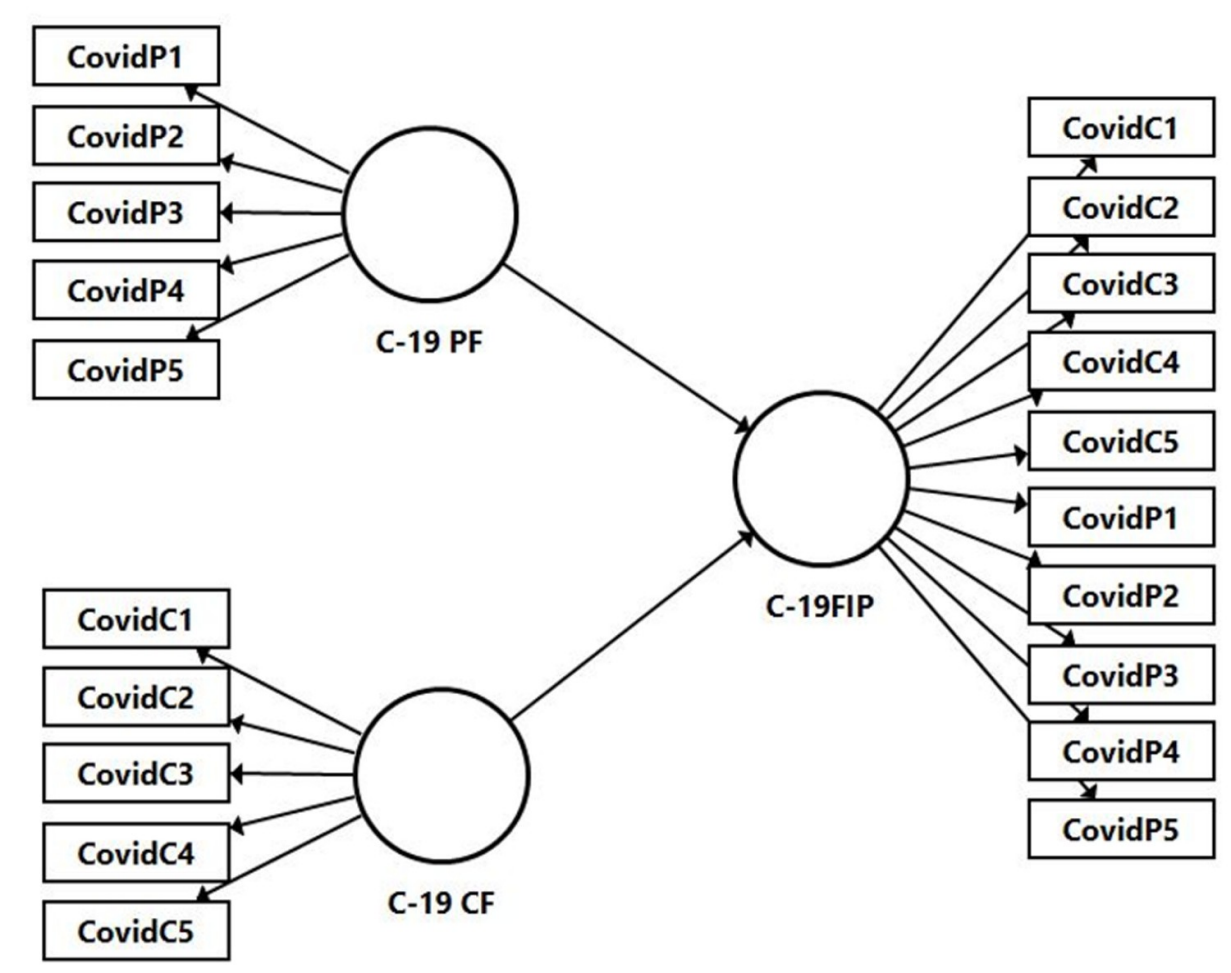
Reflective-formative second order model.

This study used the repeated indicator approach [101] to establish the reflective–formative higher-order construct of C-19 future impact perception (Fig 2). The C-19 PF and C-19 CF dimensions represent the lower-order of the more general higher-order C-19 Future Impact Perception construct. In the repeated indicators approach, all indicators of the lower-order components were assigned simultaneously to identify the higher-order component.

This study followed the suggestion of Hair et al., [80] to establish the measurement model for the second-order formative construct. In this sense, the second-order convergent validity was assessed using a redundancy analysis [102] in which the formative measured construct serves as an exogenous latent variable linked to an endogenous latent variable (i.e. a global single-item of C-19 Future Impact Perception) that measures the same concept. According to the literature, the path coefficient should assume a value equal to or greater than 0.7, i.e. the formative construct should explain at least 50% of the variance of the criterion construct [80]. In our study, the result of the redundancy analysis produced a path coefficient of 0.794 between the second-order and global single-item measurement of C-19 future impact perception, and explains 60.3% of the variance.

Next, the variance inflation factor (VIF) to evaluate the collinearity of the formative indicators was used. The VIF values for the two dimensions were less than 3 [103], indicating that collinearity was not an issue.

Bootstrapping procedures with 5000 re-sample were performed to assess the significance and relevance of the relationships between lower-order components and their higher-order components. As shown in Table 7, the outer weight for the C-19 PF and C-19 CF was significant at p< 0.001, with factor weights of 0.689 and 0.586, respectively.

**Table 7:** Validity results for formative second-order construct.

Construct	Dimensions	Std. β	Mean β	Std. Dev.	t-value	VIF
C-19 Future Impact Perception	C-19 PF	0.689	0.690	0.016	41.810**	1.05
	C-19 CF	0.586	0.586	0.019	30.696**	1.05

**p<0.001

This reveals that the first-order construct significantly explains the second-order construct. Thus, the measurement model for the second-order formative was established.

#### Assessing nomological and predictive validity

In assessing nomological validity, this study examines the relationship between the newly developed C-19 Impact Future Perception construct and Future Time Perspective Scale. By considering the cognitive theory, this study postulates that C-19 Future Impact Perception has an influence on tree dimension of Future Time Perspective.

Nurmi [28] found that the future perspective is influenced by the individual’s experiences, especially if these experiences are considered rare and salient.

**Fig 3:**
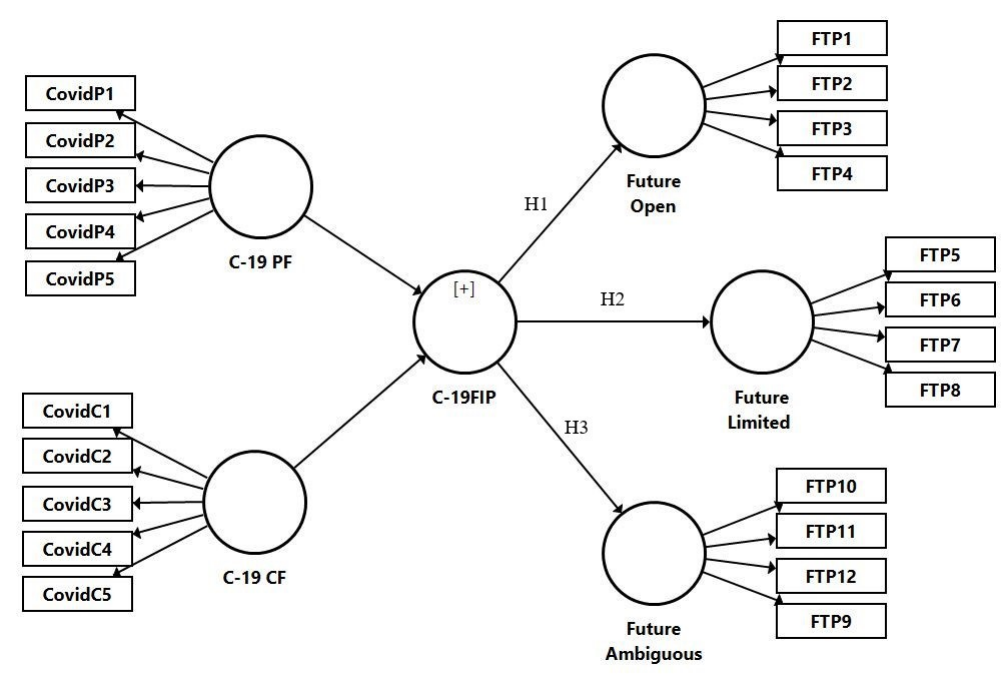
Structural model.

Therefore, the following hypotheses were proposed (Fig 3):

H1: C-19 Future Impact Perception significantly and negatively affects the perspective of the Future as Open.

H2: C-19 Future Impact Perception significantly and positively affects the perspective of the Future as Limited.

H3: C-19 Future Impact Perception significantly and positively affects the perspective of the Future as Ambiguous.

The nomological validity of the C-19 Future Perception construct was tested by predicting its relationship with Future Perspective. The reliability and validity of future perspective scale was confirmed through the values of the factor loadings, AVE, CR and CA that exceeded the recommended values of 0.70, 0.50, 0.70, and 0.80, respectively (see Table 8).

**Table 8:** Future time perspective measurement model.

Construct	Items	Loading	Cronbach’s Alpha	Rho_A	Composite Reliability	AVE
Future as Open	FTP1	0.900				
	FTP2	0.632	0.850	0.902	0.896	0.686
	FTP3	0.898				
	FTP4	0.854				
	FTP5	0.782				
Future as Limited	FTP6	0.703	0.746	0.745	0.830	0.551
	FTP7	0.809				
	FTP8	0.667				
	FTP9	0.828				
Future as Ambiguous	FTP10	0.746	0.806	0.811	0.874	0.636
	FTP11	0.886				
	FTP12	0.718				

The structural model that represents the relationship between the constructs was assessed using PLS bootstrapping with 5000 resamples to generate the values of path coefficient and their significant level (p< 0.001). Path coefficients (ß) usually vary between -1 and +1. The higher the absolute value, the stronger the predictive relationship between the constructs.

The results reveal that C-19 Future Impact Perception exerts a negative and significant effect on “Future as Open” (ß = -0.402, p< 0.001), a positive and significant effect on “Future as Ambiguous” (ß = 0.490, p< 0.01) and “Future as Limited” (ß = 0.348, p< 0.001).

In addition, this study obtained the coefficient of determination (R^2^) of 0.162 for Future as Open, 0.121 for the Future as Limited and 0.240 for Future as Ambiguous, which confirmed the impact of C-19 Future Perception on Future as Open, Future as Limited and Future as Ambiguous, thereby ensuring nomological validity.

We also evaluated the measure of effect size (f^2^) to assess whether the omitted construct has a substantial impact on the endogenous constructs. According to Cohen [104], the C-19 Future Impact Perception has a moderate effect on future as ambiguous (f^2^ = 0.31), and a little effect on future as open (f^2^ = 0.19) and future as limited (f^2^ = 0.13).

Finally, the structural model was tested for predictive relevance through the Stone–Geisser’s Q^2^ values [105, 106], using the blindfolding procedure [92]. The omission distance value should be a prime integer ranging from 5 to 10 [107] with D = 7 recommended in the literature.

According to Chin [108] Q^2^ > 0 reflect the presence of predictive relevance, while Q^2^ < 0 represents the lack of predictive relevance. The predictive relevance for endogenous variables are above zero.

Using the cross-validated redundancy approach, this study obtained Q^2^ of 0.236 for Future as Ambiguous, Q^2^ of 0.155 for Future as Open and Q^2^ of 0.116 for Future as Limited. These results supported the predictive validity of the higher-order C-19 Future Impact Perception scale.

## Discussion

The aim of this research was to develop and validate a measure to assess the impact of the COVID-19 pandemic on university students’ perceptions of their future and the country’s economy. The scale was developed using a mixed approach (inductive and deductive method) and a rigorous systematic procedure, following the recommendations of Churchill [63].

According to socio-cognitive theory [26], the environment influences the perceptions that an individual has of himself and the world, generating different behavioral responses. In this theoretical framework, particular emphasis has been given to Future Perception, especially following adverse events or perceived as uncertain. The measure developed in this study follows this theoretical approach with the aim of contributing to knowledge of the field and understanding how different factors contribute to shaping the future perceptions of university students in crisis situations.

The present study included two different samples of undergraduate students in distinct phases: Study 1 (n = 1,279), to extract the factorial structure (EFA), and Study 2 (n = 1,078), to validate the purified scale (CFA).

The results of study 1 (phase 3) showed a two-factor structure. Furthermore, the two factors indicated that the total variance of the scale was 69.77%. This result shows that both factors are representative and, therefore, the new measure has good constructive validity, indicating that C-19PIFS can be used to assess future perceptions related to COVID-19.

The first factor (C-19 PF) consists of 5 items and is related to students’ perception of the future impact of Covid-19 on work (for example: "COVID 19 will reduce my chances of finding a job").

The second factor (C-19 CF) consists of 5 items and analyzes the perceptions of the impact of Covid-19 on the economic future of the country where students live (for example: "COVID-19 will negatively affect my country’s economy"). In addition, five items were eliminated at this stage because they did not achieve a factor loading of 0.7.

This change led to the final version of the measure. The C-19 Future Impact Perception is a two-dimensional construct with 10 items and 5 response choices (from 1: “Strongly disagree” to 5: “Strongly agree”).

In the second study (phase 4) Hair’s et al. [80] recommendations were performed. At the first-order model, two different methods were used: Partial Least Square Structural Equation Modeling (PLS-SEM) and Covariance-Based Structural Equation Modeling (CB-SEM). The results show that the CB-SEM approach, while producing slightly lower values of the factor loadings than the PLS-SEM method, nevertheless generated a satisfactory fit of the two-factor model (x^2^ = 249,749 (85), p< 0.001, CFI = 0.962, TLI = 0.950 and RMSEA = 0.07). These results are in agreement with previous studies [99, 109].

In general, the two subscales (C-19 PF and C-19 CF) reached a satisfactory level in terms of Cronbach’s alpha (PLS-SEM: C-19 PF α = 0.891; C-19 CF α = 0.872; CB-SEM: C-19 PF α = 0.892; C-19 CF α = 0.865) composite reliability (PLS-SEM: CR C-19 PF = 0.920; CR C-19 CF = 0.907; CB-SEM: CR C-19 PF = 0.893; CR C-19 CF = 0.874), factor loads, AVE (PLS-SEM: AVE C-19 PF = 0.699; AVE C-19 CF = 0.662; CB-SEM: AVE C-19 PF = 0.626; AVE C-19 CF = 0.581) and Heterotrait-Montrait Ratio (HTMT = 0.247; CI [0.176–0.310]) values, confirming the authenticity of the reflective model.

The second-order model, implemented via PLS-SEM, also obtained acceptable values for VIF and significant values for the factorial weight (C-19 PF–C19FIPS = 0.689; t = 41.810; p<0.01; C-19 CF–C19FIPS = 0.586; t = 30.696; p<0.001), thus approving the second order formative model. Consequently, the theoretical and empirical evidence supports the arguments presented in this study, i.e. future perception is a higher-order multidimensional latent construct in the form of a reflective-formative type II model.

This study also confirms the nomological network and predictive validity of the superior model by showing an effect of the C-19 Future Impact Perception with the Future Time Perspective (FTP).

The multidisciplinary nature of the FTP has been emphasized in the literature [17, 36]. In the present study we have referred to three dimensions of the future time, in line with study of Brothers et al., [76]: the dimension of the “future as open”, “future as limited”, and “future as ambiguous”. Consistent with the hypotheses implemented in the present study, the effect was negative in the case of the future as open (H1: ß = -0.402, p< 0.001), while it was positive effects in the case of the future as limited (H2: ß = 0.348, p< 0.001), and ambiguous (H3: ß = 0.490, p< 0.001).

These results are in line with previous studies that have demonstrated the impact of changes and social transformations on the daily life of young people and on how they see the future [110–112], also with regard to the conviction of maintaining a job or getting the desired job (see for example, Vanhercke et al. [113]).

This appears very important considering the weight that the future assumes for the youngest. Several studies have shown that major concerns are related to the sphere of the future rather than to other domains, such as academic success [114, 115], with repercussions on mental and physical health [58–62].

Furthermore, although the current pandemic situation affects the whole world, perceptions about the future can differ profoundly between countries, based on specific conditions, such as the unemployment rate [116, 117]. Some studies have shown that there are differences in different European countries [114, 115].

For example, according to the survey conducted by Kantar [118] in October-November 2020 on the effects of the COVID-19 pandemic, 39% of European respondents said that the COVID-19 crisis has already impacted their personal income and a further 27% are expect an impact in the future.

Furthermore, although most citizens say they are uncertain about the future, citizens of Germany and Great Britain perceive less uncertainty than those living in France and Italy.

For these reasons, we have placed emphasis not only on perceptions of the future on a personal level, but also on contextual attributes (macro-level factors), recognizing the complex interaction between environment, individual and future orientation.

In general, in situations perceived as safe, when a result is particularly satisfying, personal factors exert a greater influence, while in the case of non-salient goals, context and extrinsic experiences have a greater impact in shaping the perception of the likelihood of an event occurring. Currently, little is known about how these dynamics come into play in situations perceived as uncertain and risky.

To answer this gap in the literature, we considered it appropriate to build a useful tool to foster knowledge in the field of psychological research on the "future orientation", through the analysis of the personal dimension and the nation context in situations of uncertainty (or perceived as such), as is the current pandemic. The theoretical reference model, in line with socio-cognitive theory, considers individuals and the environment as having mutually influential relationships. These evidences justify the two-dimensional nature of the new C-19FIPS measurement.

Although our article contributes to future orientation research by presenting a two-dimensional scale reliable, replicable, and theoretically informed, the present study is not without limitations that could be explored in future research. An important limitation of this study was the homogeneity of the sample which limits the generalizability of the results. The sample of the present research were university students; it would be important to evaluate the instrument with more representative samples to better understand how the scale works with subjects that have different characteristics. In addition, to explore the generalizability of the scale it might be useful to examine the role of cultural context through Hofstede’s cultural dimensions [119]. For example, the literature has shown that, in situations perceived as safe, individualistic cultures would have a higher FTP than collectivist cultures [120]: in individualistic countries it is expected that individuals take care of themselves and tend to worry about achieving their goals; in contrast, individuals in countries that have a lower individualism score expect their family members or members of a particular group to care for them in the future, devoting less personal effort to obtain the own benefits. What happens in situations perceived as adverse? Transnational and intercultural approaches could further clarify the issue.

Furthermore, this study uses a cross-sectional method for data collection. Future research should include the longitudinal evaluation of the C-19FIPS to understand how the dimensions of the C-19FIPS change and improve the assessment of the impact of COVID-19 at different points in time and solve the problem of causality.

## Conclusions

The 10-item C-19 Future Impact Perception Scale (C-19FIPS) has proven to be an acceptable and reliable tool for assessing the impact of the Covid-19 pandemic on Spanish university students’ perceptions of their future work and the economy of the country.

The scale has been subjected to numerous analyzes to confirm its reliability and validity, and is therefore suitable for use in future studies on the impact of COVID-19 in decision-making processes, especially in the Spanish context. This questionnaire can help researchers who wish to have a better overview of the impact of the pandemic in the future, but also as a useful tool for analyzing the complex relationship between psychological and environmental factors. It should also be a tool that helps the government and institutions to improve current support programs, particularly by taking young people into consideration. We believe that knowledge increases collective awareness, contributing to community decision making.

## Supporting information

S1 Appendix C-19 future perception scale. (DOC)

S2 Appendix Dataset study 1. (CSV)

S3 Appendix Dataset study 2. (CSV)
