# Supplementary material for: Development and validation of a scale to evaluate students’ future impact perception related to the coronavirus pandemic (C-19FIPS)
Source: PLoS One. 2021 Nov 19;16(11):e0260248. doi: 10.1371/journal.pone.0260248 (PMC8604316; doi:10.1371/journal.pone.0260248)
Supplement: S1 Appendix — (DOC) [file pone.0260248.s001.doc]

**S1 Appendix. C-19 Future Impact Perception Scale (C-19FIPS)**

Following the emergency situation, we are interested in understanding how the coronavirus pandemic (COVID-19) affects the future perceptions of university students.

Please read each statement carefully and tell us how much you AGREE or DISAGREE with each statement using the following rating scale. This questionnaire is completely anonymous and confidential. There are no right or wrong answers to these statements.

| Strongly Disagree | Disagree | Neither agree nor disagree | Agree | Strongly Agree |
| --- | --- | --- | --- | --- |
| 1 | 2 | 3 | 4 | 5 |

1. El COVID-19 disminuirá mis oportunidades laborales

(COVID-19 will decrease my job opportunities)

2. El COVID-19 afectará negativamente mi futuro

(COVID-19 will negatively affect my future)

3. El COVID-19 afectará negativamente a mis recursos económicos

(COVID-19 will negatively affect my financial resources)

4. El COVID-19 limitará mis posibilidades de éxito profesional

(COVID-19 will limit my chances of professional success)

5. El COVID-19 disminuirá mis opciones de encontrar trabajo

(COVID-19 will decrease my chances of finding a job)

6. El COVID-19 afectará negativamente a la economía de mi país

(COVID-19 will negatively affect the economy of my country)

7. El COVID-19 destruirá muchos puestos de trabajo en mi país

(COVID-19 will destroy many jobs in my country)

8. El COVID-19 traerá el cierre de muchas empresas en mi país

(COVID-19 will lead to closure of many business in my country)

9. El COVID-19 afectará negativamente a las micropymes en mi país

(COVID-19 will negatively affect micro enterprises in my country)

10. El COVID-19 afectará negativamente el futuro de mi país

(COVID-19 will negatively affect the future of my country)

**Scoring Algorithm:** To compute the subscales, sum the items within each subscale and divide by the number of items to compute mean scale-score values.

**C-19PF items**: mean (1, 2, 3, 4, 5).

**C-19CF items**: mean (6, 7, 8, 9, 10).
